# Epicardial tissue heating during pulsed field ablation assessed using a novel experimental model: implications for collateral damage

**DOI:** 10.1093/europace/euag166

**Published:** 2026-07-07

**Authors:** Masaharu Masuda, Hiroyuki Uematsu, Shota Kato, Hirotaka Ooka, Satoshi Kudo, Mizuki Ochi, Shin Okamoto, Kiyonori Nanto, Hata Yosuke, Masaya Kusuda, Wataru Ariyasu, Subaru Fujii

**Affiliations:** Cardiovascular Center, Kansai Rosai Hospital, 3-1-69 Inabaso, Amagasaki, Hyogo 660-8511, Japan; Cardiovascular Center, Kansai Rosai Hospital, 3-1-69 Inabaso, Amagasaki, Hyogo 660-8511, Japan; Cardiovascular Center, Kansai Rosai Hospital, 3-1-69 Inabaso, Amagasaki, Hyogo 660-8511, Japan; Cardiovascular Center, Kansai Rosai Hospital, 3-1-69 Inabaso, Amagasaki, Hyogo 660-8511, Japan; Cardiovascular Center, Kansai Rosai Hospital, 3-1-69 Inabaso, Amagasaki, Hyogo 660-8511, Japan; Cardiovascular Center, Kansai Rosai Hospital, 3-1-69 Inabaso, Amagasaki, Hyogo 660-8511, Japan; Cardiovascular Center, Kansai Rosai Hospital, 3-1-69 Inabaso, Amagasaki, Hyogo 660-8511, Japan; Cardiovascular Center, Kansai Rosai Hospital, 3-1-69 Inabaso, Amagasaki, Hyogo 660-8511, Japan; Cardiovascular Center, Kansai Rosai Hospital, 3-1-69 Inabaso, Amagasaki, Hyogo 660-8511, Japan; Cardiovascular Center, Kansai Rosai Hospital, 3-1-69 Inabaso, Amagasaki, Hyogo 660-8511, Japan; Cardiovascular Center, Kansai Rosai Hospital, 3-1-69 Inabaso, Amagasaki, Hyogo 660-8511, Japan; Cardiovascular Center, Kansai Rosai Hospital, 3-1-69 Inabaso, Amagasaki, Hyogo 660-8511, Japan

**Keywords:** Tissue hearing, Pulse-field ablation, Water-tank experiment

## Background

Pulsed-field ablation (PFA) is becoming more widely used for pulmonary vein isolation (PVI) in patients with atrial fibrillation (AF) due to its established efficacy and safety.^1–6^ Pulsed field energy theoretically offers the advantage of fewer complications due to less collateral damage compared with thermal ablation. However, temperature elevation during PFA has been demonstrated in experimental studies.^7,8^ Nevertheless, there have been limited studies examining tissue temperature elevation induced by clinically used PFA catheters. Especially, reports evaluating epicardial temperature, a key factor in assessing the risk of collateral damage such as oesophageal injury, are scarce.

The purpose of this study was to evaluate tissue temperature increase caused by commercially available PFA catheters through water-tank experiments.

## Methods

### Water-tank experiment

**Figure 1:**
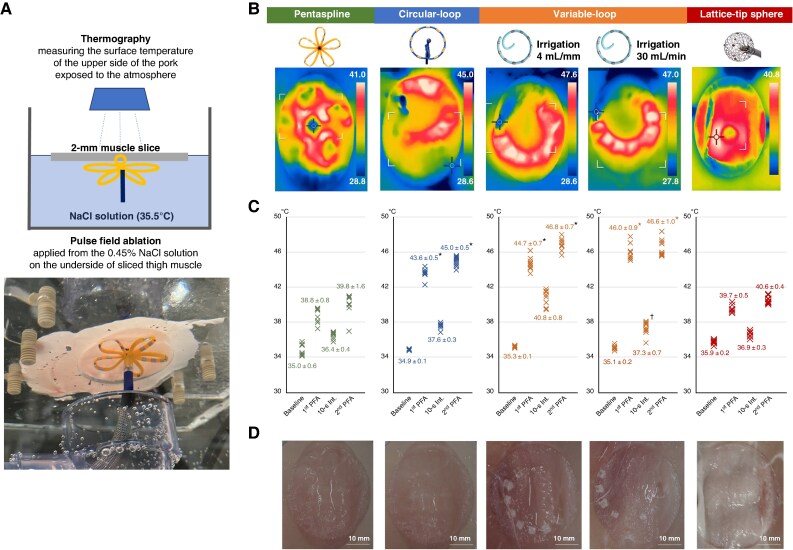
(*A*). Schematic illustration and photograph of the experimental setup. The tank was filled with half-normal saline warmed to 35.5°C. A 2-mm slice of porcine thigh muscle was fixed on the surface of half-normal saline warmed to 35.5°C. PFA was applied from beneath the slice using each catheter type, while the surface temperature on the air-exposed side was recorded by thermography. PFA, pulse field ablation. (*B*). Representative thermographic images. Temperature elevation corresponded to the electrode positions when using the single-shot PVI device. PVI, pulmonary vein isolation. (*C*) Temporal changes in surface temperature of the muscle slice on the side opposite the PFA application. The temperature elevation in pentaspline and lattice-tip spheral catheters appears to be less pronounced than that in circular-loop and carriable-loop catheters. The peak temperature was higher with the circular-loop and variable-loop catheters than with the pentaspline catheter. With the variable-loop catheter at 4 mL/min irrigation, temperature did not return to baseline during the 10-s interval between pulses. *P < 0.05 vs. pentaspline catheter. †*P* < 0.05 vs. variable-loop catheter using irrigation flow rate of 4 mL/min. (*D*) Macroscopic findings after ablation. Whitish discolouration consistent with protein denaturation was observed at the sites corresponding to the catheter electrodes only when using the variable-loop catheter, irrespective of irrigation flow rate (4 or 30 mL/min).

In the present study, we newly developed an experimental system that enables assessment of myocardial temperature elevation on the epicardial side. The tank was filled with half-normal saline warmed to 35.5°C (*Figure 1*). A 2-mm slice of porcine thigh muscle was fixed to the water surface. Pulsed field energy was applied from the saline beneath the muscle slice, and surface temperature on the upper side of the tissue exposed to air was measured using a thermal imaging camera (FLIR E85; Thermography, Osaka, Japan). PFA was delivered using three single-shot PVI catheters and a point-by-point PFA catheter: a pentaspline catheter [Farawave; Boston Scientific, Marlborough (Cambridge), MA, USA], a circular-loop catheter (PulseSelect; Medtronic, Mounds View, MN, USA), a variable-loop catheter (Varipulse; Biosense Webster, Diamond Bar, CA, USA), and a lattice-tip spherical catheter (Sphere-9, Medtronic). Each application was identical to the clinical practice (flower configulation, biphasic, bipolar 5 pulse trains for Farawave; biphasic, bipolar 4 pulse trains for PulseSelect; biphasic bipolar 3 pulse trains for Varipulse, and biphasic monopolar 4-s pulse trains for Sphere-9). To evaluate the cumulative effect of repeated applications, two applications were delivered with a 10-s interval between them. After each pair of applications, the muscle slice was replaced with a new specimen. This procedure was repeated 10 times for each catheter.

### Statistics

Continuous variables are expressed as mean ± standard deviation, and comparisons were performed using the unpaired *t*-test. Statistical analyses were conducted using commercial software (SPSS version 26.0; IBM Corp., Chicago, IL, USA).

## Results

### Heat distribution during PFA

Heat distribution on the opposite surface of the muscle slice revealed that temperature elevation was most pronounced at sites corresponding to each catheter electrode when using the single-shot PVI devices (*Figure 1*). With the lattice-tip spherical catheter, the entire catheter tip functions as an electrode and generates pulsed electric fields; consequently, heat distribution occurs in accordance with the catheter geometry.

The peak temperature was higher with the circular-loop and variable-loop catheters than with the pentaspline catheter. Notably, with the variable-loop catheter at an irrigation flow rate of 4 mL/min, temperature did not fully return to baseline during the 10-s interval. On the other hand, increasing the irrigation flow rate from 4 mL/min to 30 mL/min resulted in greater temperature reduction during the 10-s cooling period; however, it did not decrease the peak temperature during PFA application.

### Macroscopic observation

Macroscopic observation of the ablated surface of the muscle slice after two applications delivered at the predefined 10-s interval are shown in the *Figure 1*. No visible colour change on the tissue surface was observed with catheters other than the variable-loop catheter. In contrast, whitish discolouration at the electrode sites was observed only when the variable-loop catheter was used, irrespective of irrigation flow rate (4 or 30 mL/min).

## Discussion

### Tissue heating during PFA

Several studies have investigated tissue heating and irrigation-related cooling during PFA. Temperature elevation of 2.8°C at a 3-mm tissue depth has been reported based on thermocouple measurements, though metallic thermocouples are subject to antenna effects that can lead to erroneous readings.^7^ Moreover, the use of non-clinically applied focal ablation catheters and differing pulse-delivery properties limits the applicability of those results to real-world practice. Another study applied PFA to a potato model using a variable-loop catheter, showing attenuation of surface temperature rise with increased irrigation flow.^8^ However, measurement of surface temperature under irrigation does not allow accurate estimation of intra tissue temperature. In the present study, tissue temperature elevation and the whitish discolouration of the tissue surface with the variable-loop catheter was not sufficiently suppressed even when the irrigation flow rate was increased, suggesting that resistive heat–induced tissue injury could not be mitigated by increasing irrigation flow.

### Clinical implications

Tissue overheating may cause undesirable collateral damage to adjacent structures. However, the clinical implications of transient tissue heating during PFA remain unclear. Oesophageal safety with PFA has been demonstrated under clinically relevant conditions for the pentaspline catheter and the lattice-tip spherical catheter.^9,10^ However, it is possible that the risk of oesophageal injury may be higher when using the variable-loop catheters that exhibited greater tissue temperature elevation in this study than the pentaspline or the lattice-tip spheral catheters. Although higher irrigation flow may help prevent thromboembolic events, the potential risk of collateral damage must still be carefully considered. Further clinical data and analyses focusing on the potential influence on collateral injury are warranted for each catheter type.

### Limitation

A limitation of this study is that the temperature measured on the side opposite to the PFA application in a porcine thigh muscle slice does not necessarily reflect the deep tissue temperature during clinical use. This discrepancy may be attributed to differences between human myocardial tissue and porcine skeletal muscle, the use of half-normal saline during ablation, as well as the absence of blood perfusion in the experimental model. The efficacy and safety in clinical use should be determined based on data obtained from animal experiments that more closely simulate human application and from clinical studies.

## Conclusion

The extent of epicardial tissue overheating during PFA differed considerably among devices. In particular, substantial epicardial temperature elevation was observed with the variable-loop catheter regardless of irrigation flow rate. As this study was conducted using a water-tank experimental model, the clinical implications of these findings are unclear. Further device-specific clinical validation is warranted to assess safety related to collateral damage.

## Data Availability

The data underlying this article will be shared on reasonable request to the corresponding author.
